# A Comparison of Accuracy of Different Dental Restorative Materials between Intraoral Scanning and Conventional Impression-Taking: An In Vitro Study

**DOI:** 10.3390/ma14082060

**Published:** 2021-04-19

**Authors:** Jung-Hwa Lim, Utkarsh Mangal, Na-Eun Nam, Sung-Hwan Choi, June-Sung Shim, Jong-Eun Kim

**Affiliations:** 1 Oral Research Science Center, Department of Prosthodontics, Yonsei University College of Dentistry, Seoul 03722, Korea; erin850313@gmail.com (J.-H.L.); jennynam90@yuhs.ac (N.-E.N.); 2 Institute of Craniofacial Deformity, Department of Orthodontics, Yonsei University College of Dentistry, Seoul 03722, Korea; utkmangal@yuhs.ac (U.M.); selfexam@yuhs.ac (S.-H.C.); 3 Department of Prosthodontics, Yonsei University College of Dentistry, Yonsei-ro 50-1, Seodaemun-gu, Seoul 03722, Korea; jfshim@yuhs.ac

**Keywords:** dental restorative materials, intraoral scanner, CAD/CAM, digital impression, accuracy, powder spray, crown substrate

## Abstract

The properties of underlying substrates influence the quality of an intraoral scan, but few studies have compared the outcomes using common restorative materials. In this study, we aimed to compare the accuracy of digital and conventional impressions recorded for four different dental materials as the substrates. Experimental crowns were produced with a metallic surface (gold or cobalt-chromium alloy (Co-Cr)) or without a metallic surface (zirconia or PMMA (polymethyl methacrylate)). A conventional impression was made in the conventional group (CON group), and gypsum models were subsequently scanned with a tabletop scanner. An intraoral scanner was used to scan the crowns either after applying a powder spray to reduce the surface reflectivity (IOS-P group) or without the powder spray (IOS group). The scans were assessed in three dimensions for precision and trueness. The accuracy did not differ between the CON and IOS groups for the non-metallic crowns. However, it was statistically different for the Co-Cr metallic crown, reducing trueness observed between groups as CON > IOS > IOS-P. The study evidences the differences in outer surface accuracy observed with a change in the substrate material to be imaged using an oral scanner and with the impression method. These findings suggest that the restoration material present in the oral cavity should be considered when selecting an impression-taking method.

## 1. Introduction

A dental impression is defined in the Glossary of Prosthodontic Terms as “a negative imprint or a positive digital image display of intraoral anatomy; used to cast or print a three-dimensional (3D) replica of the anatomic structure that is to be used as a permanent record or in the production of a dental restoration or prosthesis” [1]. Accurate impression acquisition promotes successful dental restorations [2]. Most conventional materials used for impression acquisition are irreversible hydrocolloids or elastic dental materials such as vinyl siloxane ether, vinyl polysiloxane, and polyether. These materials are biocompatible and provide high accuracy for conventional impression-acquisition methods [3]. However, the absence of a standard operating protocol for the impression taking procedure and deformation of the impression or the plaster cast material tends to adversely affect the model accuracy, consequently affecting the accuracy of the three-dimensional (3D) model data and producing fixed prostheses [4]. Moreover, the conventional impression-making procedure is often reported to be uncomfortable and unpleasant due to it eliciting the gagging reflex and the disagreeable taste of the impression material [5].

Rapid technological advancements in computer science have produced a paradigm change in fabrication protocols. Computer-aided design (CAD) and computer-aided manufacturing (CAM), along with digital intraoral scanners, are increasingly being applied for producing dental prostheses and models [6]. Compared with the conventional methods, acquiring a digital impression using an intraoral scanner has the advantages of easy repetition, direct model visualization, improved time efficiency, and chairside data acquisition for CAD/CAM prosthetics. Patients also show a strong preference for digital impression acquisition over the conventional methodology in the clinical setting [7]. The digital technique also plays a significant role in treatment planning via simulation in the aesthetic area. Direct model visualization and a preview of the final occlusion and smile design can enhance the overall treatment outcome. The ad hoc integration of morphometric software also helps when comparing the overall accuracy of the prosthesis, especially concerning the marginal adaptations such as for crowns and abutments [8].

Technically, digital impressions can be integrated with other digital datasets, such as those obtained using cone-beam computed tomography (CBCT) and optical facial scans [9], and therefore the accuracy of intraoral scan data for reproducing various oral environments very important. Hence, recent studies comparing conventional and direct intraoral scanning have consistently concluded that using direct intraoral scanners is an acceptable alternative to conventional methodologies [10,11,12,13].

The preference for directly digitalized intraoral impressions has led to an equivalent growth in studies focused on the technical robustness between different intraoral scanners and indirect digitization protocols using a desktop scanner. In their extensive work, Guth et al. [14] examined the quality of scans produced with five sets of direct digitizing scanners. The authors studied spatial divergences and observed significant differences between direct digitalizing scanners, as well as with indirect cast digitization. Although a standard metal-based (titanium) reference was used, the authors could not conclusively recommend powder use prior to scanning and recommended further assessment. Similar work was also conducted by Tomita et al. [15] who used a standard epoxy model and compared digitalization of the plaster models generated with commonly used alginate and silicone-based impressions. With a unique coordinate system-based approach, their findings supplemented the observations of earlier studies. Additionally, they concluded a tendency of intraoral scanning processes to show negative deviations (i.e., being smaller than the set reference). In the same study, indirect digitization showed a positive deviation. However, they suggested that the intraoral scanning process may be more accurate than conventional plaster model methods for evaluating digital linear distance.

However, the results obtained using intraoral scanners have been affected by differences in the restorative materials and variations in tissue surfaces due to the nonuniform scattering of light [16]. Li et al. [17] reported that using an intraoral scanner to scan materials with high translucency resulted in lower scan accuracy along with morphological variations. Bocklet et al. [18] reported that both the type of restoration material and the type of intraoral scanner affected the accuracy of the scan data. To overcome some of these challenges in direct digital scanning, spraying on powder before scanning has been recommended [12,14], but a standard protocol is lacking, and the results differ with the scanning process employed.

When manufacturing a prosthesis, it is necessary to consider both information about the abutment teeth and the relationships with the surrounding teeth. In a recent typodont study, Son et al. [19] evaluated the impact of interproximal distance on scan accuracy to record tooth preparations, concluding that trueness of a digitalized impression improved with the interproximal distance (1.0 > 0.6 mm). These observations were believed to impact access to light from the scanner on the contour of the adjacent tooth, where sharp and angled surfaces lead to a false bridging-like effect. While the underlying surface geometry is significant, the discrepancy in optical interaction could also play a significant role and might warrant an algorithmic adjustment. Clinically, patients can present with translucent or opaque dental materials depending on their treatment history.

A recent systematic review concluded that the accuracy of digital workflow for a fixed dental prosthesis was similar to conventional protocols. It was emphasized that the quality of the scanned data was critical for determining the accuracy of the additively manufactured casts [20]. Therefore, the validity of a digital impression can be questioned, particularly in cases with multiple types of restorations and prosthetics. The quality of the data obtained may vary with the shape and surface characteristics of prosthetic materials present in the adjacent and opposing teeth.

Moreover, a recent study investigated the internal fitting of a prosthesis using an intraoral scanner [21]; however, in the present literature, there are limited studies on the effect of the relationship between the external fitting such as proximal and occlusal contact and the surrounding material (tooth or restoration) from a clinical perspective; thus, leaving a lacuna in knowledge about the possible impact of material in the immediate vicinity. In an attempt to address this issue, we hypothesized that when using an intraoral scanner, the accuracy of the final prosthesis is affected by the material properties of an adjacent or opposing prosthetic restorative material. Therefore, we aimed to study the effect of restorative materials on the reproduction of the exterior surfaces.

This study was designed to compare the accuracies of digital impressions of crowns manufactured with polymethyl methacrylate (PMMA), zirconia (Zr), gold, and cobalt-chromium alloy (Co-Cr) using an intraoral scanner or a conventional impression acquisition method. The following null hypotheses were addressed: (1) no differences exist in precision and trueness among crowns fabricated of different dental restorative materials produced using a digital impression, (2) no significant differences exist between conventional and digital methods of impression taking with different materials, and (3) the scan quality does not differ between applying and not applying powder.

## 2. Materials and Methods

### 2.1. Fabrication of the Experimental Model

An abutment preparation model of the first maxillary left molar (A55SA231/262, Nissin Dental Products, Kyoto, Japan) was used as the experimental model in this study. This model was scanned using a tabletop scanner (Identica T500, Medit, Seoul, Korea). The obtained file was converted to stereolithography (STL) format, and the crown was designed using dental CAD software (Exocad Dental CAD, Exocad, Darmstadt, Germany). The design file was used to produce crowns from the following four different materials: CAM produced non-metal; Zr (KATANA Zirconia STML, Kuraray Noritake Dental, Tokyo, Japan) and PMMA (Vipi block, Vipi, Sao Paulo, Brazil) crowns; and metal alloy crowns (gold alloy and Co-Cr alloy) were produced by milling wax discs (Mazic CAD/CAM wax, Vericom, Gangwon-do, Korea) using CAM (M5, Zirkonzahn, Bolzano, Italy), with each experimental crown, then, produced using a casting process. The four types of crowns were finished by applying a clinical polishing protocol (Figure S1). Information about the scanner systems used in this study is provided in Table 1.

### 2.2. Digitization of the Experimental Models

Each experimental crown was directly scanned using a tabletop scanner to obtain the reference scan data. The powder was sprayed on the crown to reduce the effects of reflections from the crown surface before scanning. A conventional impression was digitized (CON group) using the following process: First, eight impressions were made on the experimental crown for each crown material using Vinyl Polysiloxane (PVS) (exafine regular and putty, GC, Tokyo, Japan). Second, a gypsum model was produced and scanned once using the same tabletop scanner (T500). The data were obtained in the STL format (*n* = 8) for each crown material. In addition, data were similarly acquired to conduct a preliminary experiment using an intraoral scanner (TRIOS 3, 3shape, Copenhagen, Denmark). The 3D scan data for the gypsum model from both scanners (tabletop and intraoral) were sequentially compiled and compared to evaluate the difference in accuracy between the two scanners.

An intraoral scanner was used to scan the individual crowns constructed from the four different materials, mimicking the clinical digital impression acquisition protocol. The experimental model was scanned eight times for each material using the intraoral scanner. The clinical scanning protocol often includes spraying on a powder prior to scanning. In order to eliminate the confounding factor, we performed scans in two separate groups, i.e., with powder (EzScan, DMAX, Daegu, Korea) (IOS-P group) and without powder (IOS group) (Figure S1).

### 2.3. Three-Dimensional Accuracy Analysis

The precision and trueness of the conventional and direct digital impression methods were assessed based on the ISO 5725-1 standard [22].

The scan data with different coordinates obtained from each group were viewed using 3D morphometric analysis software (Geomagic Control X, 3D Systems, Rock Hill, SC, USA) and aligned with the reference data. The best-fit algorithm was used for comparing the scanned data files following the optimal alignment of the meshes. The ability to superimpose the data varied with the analysis method. The deviation between reference and test group scan data was calculated as the root mean square (RMS) error:

(1) $$RMS=\frac{1}{\sqrt{n}}\sqrt{\overset{n}{\underset{i=1}{\sum }}{\left(\chi_{1,i}-\chi_{2,i}\right)}^{2}}$$

where $\chi_{1,i}$ is the measurement point of reference *i*, $\chi_{2,i}$ is the measurement point of scan data *i*, and *n* is the total number of points measured in each dataset. The overall deviations were presented as color maps to facilitate intuitive comparisons, with deviations displayed from –500 to +500 µm, and values from –10 to +10 µm displayed in the same color.

**Figure 1:**
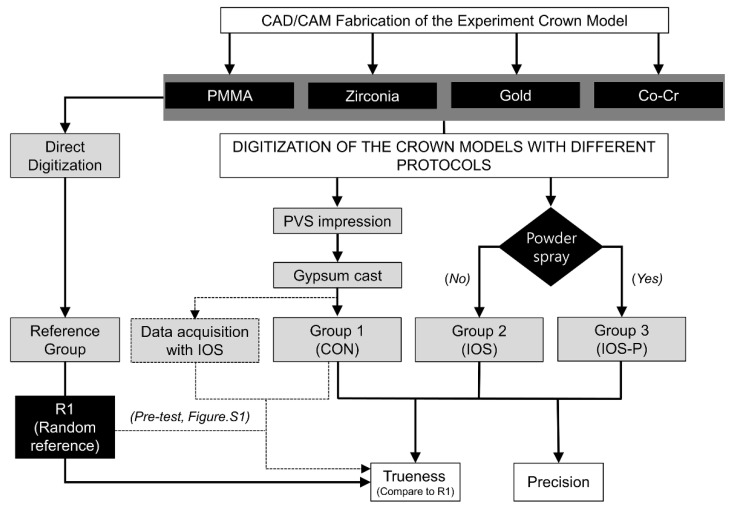
Flow chart of the study design for comparing accuracies between the two impression-taking protocols. Eight impressions were recorded for each material in accordance with the methodology of each group. CAD, computer-aided design; CAM, computer-aided manufacturing; CON, conventional; IOS, intraoral scan; and IOS-P, intraoral scan with powder.

To evaluate precision, the data obtained by repeatedly imaging each crown material (eight times per group) were superimposed in a pairwise manner to calculate the mean value of the 3D RMS error. The trueness was evaluated by superimposing an STL file from a reference scan against the data from individual experimental groups (*n* = 8), as summarized in Figure 1.

Additionally, for the IOS group, the distance deviations of the corresponding models for the different material groups were displayed with color maps so that areas of good and poor agreement could be identified.

### 2.4. Statistical Analysis

The Mann–Whitney test was used to statistically compare the different materials for the same impression-taking protocol. The Kruskal–Wallis test, which is not affected by the distribution and is a nonparametric procedure, was applied to detect intergroup differences in the average distances (RMS errors). The Bonferroni correction method was used (α = 0.017) for post hoc testing. All calculations were performed using standard statistical software (version 25.0, SPSS Statistics, IBM Corporation, Chicago, IL, USA).

## 3. Results

Morphometric differences between the samples were calculated based on the use of different prosthetic materials using conventional and digital impression-taking protocols. The recorded RMS error was tabulated to compare the accuracy between the non-metallic, including PMMA and Zr (Table S1), and metallic, including gold and Co-Cr, materials (Table S2). A lower RMS error value indicated better precision and trueness.

**Figure 2:**
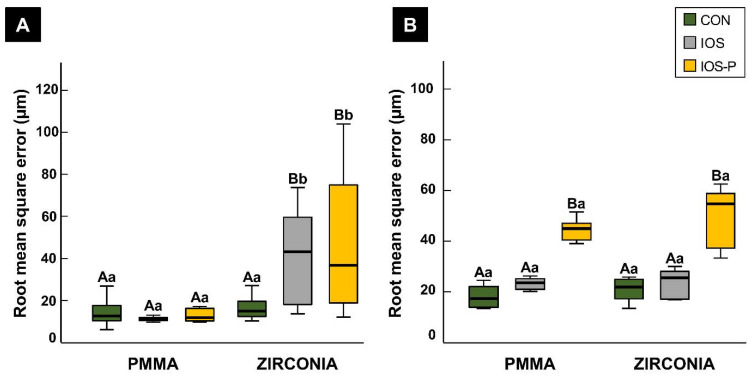
Comparison of non-metallic restorative materials using different impression-taking protocols. (**A**) Precision; (**B**) trueness. Different uppercase letters indicate significant differences between groups within a category on the x-axis. Different lower-case letters indicate significant differences between two materials for the same impression-taking protocol. Each box plot shows the median, first and third quartiles, and range. CON, conventional; IOS, intraoral scan; and IOS-P, intraoral scan with powder.

The different materials were grouped into metallic and non-metallic types, and the scan accuracy was evaluated. The 3D analysis comparing the non-metallic materials revealed a significant difference (*p* < 0.001) between scans of the PMMA and Zr obtained using an intraoral scanner with and without the use of powder, with PMMA showing better precision (IOS mean 11.19 μm and median 11.30 μm and IOS-P mean 15.75 μm and median 11.80 μm). The precision was significantly better (*p* < 0.001) in the CON group (mean 16.42 μm and median 14.95 μm) than in the IOS group (mean 40.16 μm and median 43.20 μm) for Zr but not for PMMA. In contrast to Zr, the PMMA samples showed good precision values with no significant difference between the scan groups (Figure 2A).

The trueness did not differ significantly (*p* > 0.05) between the two non-metallic materials in the CON and IOS groups. However, the RMS errors were significantly higher in the IOS-P group than in the CON and IOS groups, for both PMMA and Zr, as shown in Figure 2B.

**Figure 3:**
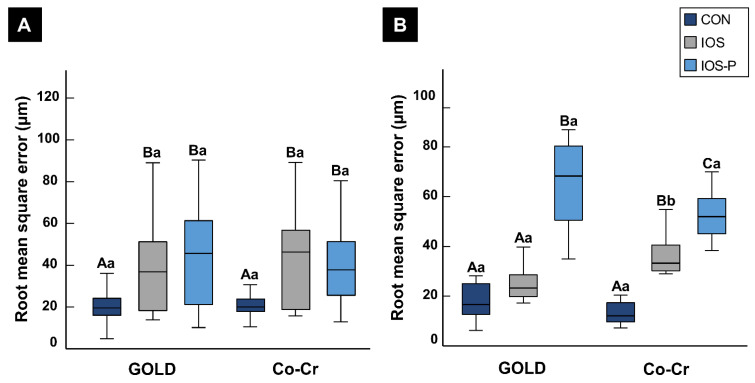
Comparison of metallic restorative materials using different impression-taking protocols. (**A**) Precision; (**B**) trueness. Different uppercase letters indicate significant differences between groups within a category on the x-axis. Different lower-case letters indicate significant differences between two materials for the same impression-taking protocol. Each box plot shows the median, first and third quartiles, and range. CON, conventional; IOS, intraoral scan; and IOS-P, intraoral scan with powder.

The precision values showed similar patterns for the metallic materials (gold and Co-Cr). The precision was significantly better (*p* < 0.05) in the CON group than in the IOS and IOS-P groups. A comparison of the IOS and IOS-P groups revealed that the precision for Co-Cr was better in the IOS-P group, with lower mean and median RMS errors of 38.95 μm and 37.75 μm, respectively, whereas for gold, the RMS errors (mean 43.59 μm and median 45.70 μm) were lower in the IOS group. However, no significant differences were observed between the materials with and without the application of powder, as shown in Figure 3A.

The trueness analysis between the metallic materials showed that the accuracy was better in the CON group than in the other groups. The RMS errors in scans of gold experimental crowns showed significant differences (*p* < 0.001) with powder application in the IOS-P group. In contrast, there were no significant differences (*p* > 0.05) in average values for the IOS group (mean 25.06 µm and median 23.25 µm) and the CON group (mean 17.91 µm and median 16.65 µm).

In contrast to gold crowns, the trueness for Co-Cr crowns was worse in the IOS and IOS-P groups than the CON group, with significant differences between the three scanning protocols (*p* < 0.001), as shown in Figure 3B.

**Figure 4:**
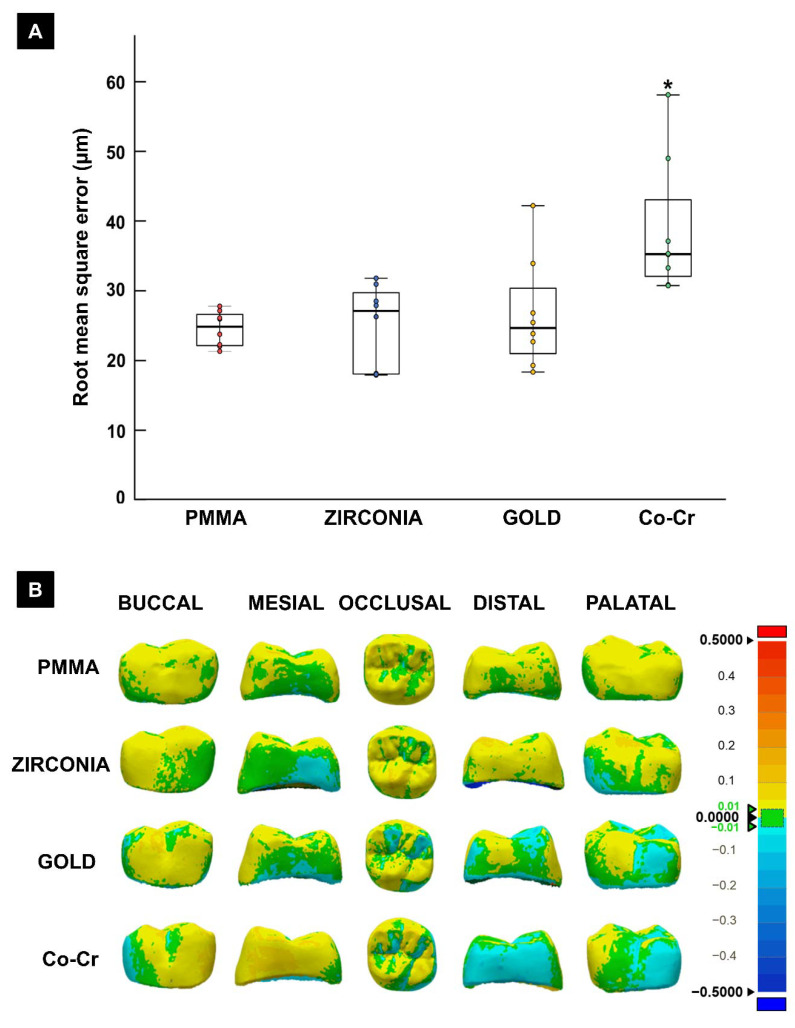
(**A**) Trueness comparison between different restorative materials using the intraoral digital impression-taking protocol. The asterisk indicates a significant difference from the other groups (*p* < 0.05); (**B**) Color maps showing the outcome of morphometric comparison, with positive deviations (larger than reference) and negative deviation (smaller than reference) between the materials for the different crown surfaces.

The accuracy analysis of the digital impression-taking protocol using an intraoral scanner revealed no significant differences in the average values between PMMA (mean 23.15 µm and median 23.45 µm), Zr (mean 25.06 µm and median 25.55 µm), and gold (mean 36.50 µm and median 23.25 µm). However, the trueness was significantly worse for Co-Cr (*p* < 0.05) than for the other three materials (Figure 4).

## 4. Discussion

In this study, we analyzed how different materials influence the key step of making a digital impression. In the traditional method of impression taking, deformation of the impression material and associated laboratory procedures decrease the accuracy of the result. A digital impression has the advantage of simplifying the process by reducing the accumulated error. However, digital impressions, obtained using a scanner, are affected by the surface physical properties of the object due to differences in how they reflect light [12]. Such differences in optical properties between the tooth and dental materials can affect the accuracy of the scan. In this study, we found significant differences between impressions obtained using conventional and digital protocols. There were also further differences depending on the crown fabrication material. Therefore, the null hypotheses of this study were rejected.

Precision refers to the closeness of measured values and represents a metric of the repeatability of the measurements, whereas trueness reflects how far the measured value is from the true or actual value of the object being measured [22]. A gypsum model of each material type was assessed for trueness using a tabletop scanner and intraoral scanning as a preliminary experiment. The observations for trueness were consistent with the research finding of Ender et al. [11] that trueness was better for the conventional method than that of an intraoral scanner, as indicated by mean errors of 7.0 and 18.4 μm, respectively. In addition, the present study found no significant differences in trueness between the four different material crowns, digitalized with conventional impression-taking methods. (Table S3). This could be attributed to the absence of optical variables such as translucency in digitized samples fabricated using the conventional impression-taking technique. It is also consistent with Kurz et al. [23] who found that a higher translucency of the materials and a larger scanning angle reduced the overall accuracy. However, the above observations were consistent with a study in the literature that reported that the scanning accuracy of plaster models was higher for extraoral scanners than that for intraoral scanners [24], although from a clinical perspective, there was no established workflow involving scanning of gypsum models with an intraoral scanner. The results in Figure S2 make it easy to understand the dimensional changes that can occur using a scanner and those associated with the impression material and errors during laboratory procedures. These results have identified an error range of an intraoral scanner that could be utilized as a reference standard when evaluating the trueness of scans of crowns constructed from various materials.

Previous studies have found the internal fit values of crowns fabricated using a digital impression acquisition method to be similar to or better than that using the conventional method, supporting the use of prostheses fabricated with digital techniques [25,26]. The adaptation and fit of the prosthetic crown are guided by its relationships with the adjacent and opposing teeth, which are facilitated by the master cast [27]. Direct intraoral scanning replaces a physical master model with a virtual setup, and therefore necessitates reproducible relationships with the teeth adjacent to the abutment site. Intraoral scanners are inherently affected by the dispersion of light and its uniformity, and therefore the surface properties of the surrounding teeth and restorations can influence scanning outcomes. There have also been previous reports of the impact of the translucency of natural tissues, with scan accuracy being lower for enamel than for dentin [18].

Since the impact of commonly used clinical restorative material has been underreported, we analyzed the effects of different non-metallic and metallic materials between the two protocols for intraoral impressions. Furthermore, since previous studies have advocated spraying on powder prior to performing an intraoral digital scan in order to improve the capture quality [28,29], we included an additional third scanning group comprising powder-sprayed crown materials.

The accuracy, using non-metallic materials (PMMA and Zr), was similar for the two digitization protocols. In contrast, the precision was markedly lower for Zr crowns using an intraoral scanner, indicating poor dimensional repeatability between successive scans. In addition, it can be suggested that the presence of a PMMA-based fixed dental prosthesis [30] does not significantly influence the accuracy of an arch scan. These findings differ from the conclusions of Dutton et al. [31], who found a trend of the accuracy being lower for materials with higher translucency. Substrate-dependent differences were also reported by Michelinakis et al. [32] for comparisons of the accuracy of a full arch scan using an intraoral scanner. The studies by Ender et al. [11] and Renne et al. [33] found marked differences in trueness according to the substrate used, i.e., while Ender et al. reported a mean precision of 57.4 µm for a ceramic substrate scan, Renne et al. found a mean precision of 105.6 µm for a full-arch PMMA restoration. The extent of the scan and the arch geometry are also influencing factors [34]. However, these previous researchers agreed that the substrate influenced the accuracy of an impression, which was consistent with our findings. Although our study was limited to a single crown, significant differences in precision values were found between PMMA (11.19 ± 0.99 µm, mean ± SD) and Zr (40.16 ± 21.48 µm) crowns. Furthermore, spraying on powder before scanning decreased the quality of scans in the non-metallic groups, which contrasted with previous findings. For example, Lee et al. [35] investigated a PMMA master model replica of a maxillary first molar and found that applying powder improved both the precision and trueness of the scans.

The polished gold and Co-Cr surfaces for the metallic crowns resulted in higher quality images and higher repeatability in the CON group than in the IOS group, thereby validating the scanning accuracy. These variations in trueness and precision during intraoral scanning indicate the possibility of significant deviations in the dimensions and fit of a crown when there is an opposing or adjacent metallic restoration. Interactions with metallic intraoral components also occur in the presence of bonded orthodontic appliances. Recent studies that compared the impact of different bracket materials also found that metallic structures influence scan accuracy. For example, Kang et al. [35,36] observed a discrepancy of 300 µm in the first molar region in the presence of brackets, while Song and Lim [37] reported a maximum discrepancy of up to 1.5 mm with effects in the interdental region. This means that the presence of adjacent or opposing metallic restorations could influence the virtual setup of a crown preparation. This theory is also supported by the cadaveric study by Bocklet et al. [18], who found that the presence of a metallic amalgam restoration adversely impacted the overall scan accuracy.

Due to the presence of metallic surfaces tending to increase errors when using an intraoral scanner, previous studies [17,23] have recommended the use of powder to overcome the associated challenges of surface moisture and angular reflections. Although the powder thickness can vary between operators, a scanning software algorithm can be used to compensate for these differences [38,39,40]. In contrast, the present study found a marked reduction in trueness when powder was sprayed onto either gold or Co-Cr crowns. In addition, the mean precision values for the Co-Cr crowns were not significantly affected by applying powder. The principal determinant of scanner accuracy is the optical principles underlying image acquisition and processing, with powder used only as an adjunct [38]. Therefore, the contrasting findings with the prescan powder application, an adjunctive step, cannot be considered to be a causative factor.

The above-mentioned observations suggest that errors are likely when performing a direct intraoral digital scan of an arch segment with existing metallic prostheses, which could impact the overall outcome of the planned dental prosthesis. The errors were visualized by qualitatively observing the deviations among the four materials presented as color maps. PMMA crowns, showed good image reproducibility using an intraoral scanner; however, Co-Cr crowns showed an increased discrepancy between the different opposing faces, namely the mesial-distal and buccal/lingual directions.

The present study performed experiments using an in vitro setup, and hence was not affected by space constraints with clear access to the crowns in the IOS group. In contrast, in the clinical situation, the intraoral condition presents substantial accessibility restrictions that might lead to inadvertent errors, especially for posterior teeth. The impact of such an access constraint was also discussed in the typodont study by Son et al. [19], noting a loss of precision due to scanner light access. While our study design, comprised of a single crown setup, avoids such access limitation and permits an objective comparison of the substrates, the interpretation of the clinical setup results is constricted.

Therefore, it can be concluded that the crown surfaces impact the accuracy to varying degrees that differ markedly with the underlying substrate material, which is consistent with the conclusion of Dutton et al. [31]. However, the observations made in the present study might not be directly transferable to a clinical environment since variables such as the spatial relationship of the abutment, the presence of proximal teeth, and saliva in the mouth were not evaluated. Hence, replicating this study in an oral environment may produce different results, using a framework for follow-up in vivo research. Additionally, in contrast to the present results, several previous studies have found that spraying powder before oral scanning can improve the accuracy [41,42]. This discrepancy might be attributable to the use of different scanning technologies. The current study used a single scanner with a specific operating principle. Further studies with different intraoral scanner systems and surface materials need to be performed.

## 5. Conclusions

The digitization accuracy of an impression is significantly better for the conventional crown fabrication protocol than for the direct intraoral scanning method for metal-based materials. Gold and Co-Cr crowns show lower accuracy associated with negative deviations in the morphometric analysis.

## Figures and Tables

**Table 1:** Scanning systems used in the study.

	Tabletop Scanner	Intraoral Scanner
Manufacturer	Medit	3shape
Product name	Identica T500^®^	TRIOS 3^®^
Scan method	Structured illumination	Confocal
Light source	Blue light	Light-emitting diode
Acquisition method	Still imaging	Video
Powder use	Required	Not required

## Supplementary Materials

The following are available online at https://www.mdpi.com/article/10.3390/ma14082060/s1, Figure. S1: Fabricated experimental models and digitization protocols, Figure S2: Comparison of accuracy between the tabletop scanner and intraoral scanner, *p* < 0.001, Table S1: Accuracy comparison of non-metallic restorative materials with different impression acquisition methods (RMSE, µm), Table S2: Accuracy comparison of metallic restorative materials with different impression acquisition methods (RMSE, µm), Table S3: Accuracy comparison of conventional impression with different materials (RMSE, µm).
