# Supplementary material for: A Comparison of Accuracy of Different Dental Restorative Materials between Intraoral Scanning and Conventional Impression-Taking: An In Vitro Study
Source: Materials (Basel). 2021 Apr 19;14(8):2060. doi: 10.3390/ma14082060 (PMC8073328; doi:10.3390/ma14082060)
Supplement: Supplementary file 1 [file materials-14-02060-s001.zip › materials-1151795-supplementary.pdf]

materials
MDPI

Supplementary Materials

A Comparison of Accuracy of Different Dental Restorative Materials between Intraoral Scanning and Conventional Impression-taking: An In Vitro Study

Jung-Hwa Lim [1], Utkarsh Mangal [2], Na-Eun Nam [1], Sung-Hwan Choi [2], June-Sung Shim [3] and Jong-Eun Kim [3,*]

[1] Oral Research Science Center, Department of Prosthodontics, Yonsei University College of Dentistry, Seoul 03722, Korea; erin850313@gmail.com (J.-H.L.); jennynam90@yuhs.ac (N.-E.N.)
[2] Institute of Craniofacial Deformity, Department of Orthodontics, Yonsei University College of Dentistry, Seoul 03722, Korea; utkmangal@yuhs.ac (U.M.); selfexam@yuhs.ac (S.-H.C.)
[3] Department of Prosthodontics, Yonsei University College of Dentistry, Yonsei-ro 50-1, Seodaemun-gu, Seoul 03722, Korea; jfshim@yuhs.ac
* Correspondence: gomyou@yuhs.ac (J.-E.K.); Tel.: +82-2-2228-3166

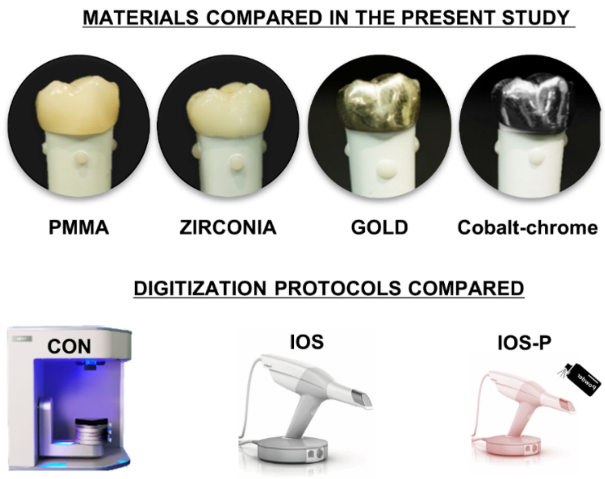

Figure S1. Fabricated experimental models and digitization protocols.

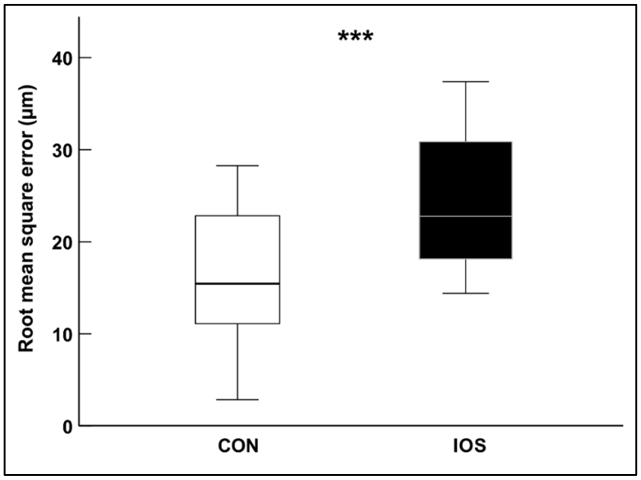

Figure S2. Comparison of accuracy between the table top scanner and intraoral scanner. P< 0.001.

Table S1. Accuracy comparison of non-metallic restorative materials with different impression acquisition methods (RMSE, μm).

	CON			IOS			IOS-P		
Materials	Mean ± SD	Median	IQR	Mean ± SD	Median	IQR	Mean ± SD	Median	IQR
Precision									
PMMA	13.95 ± 5.09	12.50 Aa	7.38	11.19 ± 0.99	11.30Aa	1.53	15.75 ± 13.71	11.80 Aa	6.05
Zirconia	16.42 ± 5.69	14.95 Aa	8.05	40.16 ± 21.48	43.20 Bb	42.08	46.12 ± 30.22	36.75 Bb	57.38
Trueness									
PMMA	18.03 ± 4.40	17.25 Aa	8.83	23.15 ± 2.37	23.45 Aa	4.50	44.40 ± 4.29	44.90 Ba	7.53
Zirconia	20.90 ± 4.78	21.90 Aa	8.50	23.53 ± 5.64	25.55 Aa	11.60	49.65 ± 12.06	54.70 Ba	23.65

The different uppercase letters indicate significant differences within the group. The lowercase letters show statistical differences between the two materials with same impression protocol. Same small and large script letters indicate no significant differences in horizontal row and vertical column (p< 0.05). IQR, interquartile range (q3-q1).

Table S2. Accuracy comparison of metallic restorative materials with different impression acquisition methods (RMSE, μm).

	CON			IOS			IOS-P		
Materials	Mean ± SD	Median	IQR	Mean ± SD	Median	IQR	Mean ± SD	Median	IQR
Precision									
Gold	19.61 ± 6.76	19.50 Aa	8.35	38.68 ± 20.87	36.90 Ba	34.03	43.59 ± 23.26	45.70 Ba	42.23
Co-Cr	21.01 ± 6.01	20.10 Aa	6.60	40.62 ± 22.71	46.35 Ba	38.73	38.95 ± 17.30	37.75 Ba	26.63
Trueness									
Gold	17.91 ± 7.68	16.65 Aa	13.85	25.06 ± 7.50	23.25 Aa	11.33	65.01 ± 18.67	68.25 Ba	33.58
Co-Cr	13.24 ± 4.75	12.10 Aa	9.08	36.50 ± 9.18	33.25 Bb	13.73	52.60 ± 10.34	51.95 Ca	17.15

The different uppercase letters indicate significant differences within the group. The lowercase letters show statistical differences between the two materials with same impression protocol. Same small and large script letters indicate no significant differences in horizontal row and vertical column (p< 0.05). IQR, interquartile range (q3-q1).

Table S3. Accuracy comparison of conventional impression with different materials (RMSE, μm).

	Precision			Trueness		
Materials	Mean ± SD	Median	IQR	Mean ± SD	Median	IQR
PMMA	13.95 ± 5.09	12.50 Aa	7.38	18.03 ± 4.40	17.25 Aa	8.83
Zirconia	16.42 ± 5.69	14.95 Aa	8.05	20.90 ± 4.78	21.90 Aa	8.50
Gold	19.61 ± 6.76	19.50 BCa	8.35	17.91 ± 7.68	16.65 Aa	13.85
Co-Cr	21.01 ± 6.01	20.10 Ca	6.60	13.24 ± 4.75	12.10 Aa	9.08

Same uppercase letters indicate no significant differences in vertical column (p< 0.0083). IQR, interquartile range (q3-q1).
